# Weight loss treatment for COVID-19 in patients with NCDs: a pilot prospective clinical trial

**DOI:** 10.1038/s41598-024-61703-1

**Published:** 2024-05-14

**Authors:** Kuat Oshakbayev, Aigul Durmanova, Zulfiya Zhankalova, Alisher Idrisov, Gulnara Bedelbayeva, Meruyert Gazaliyeva, Altay Nabiyev, Attila Tordai, Bibazhar Dukenbayeva

**Affiliations:** 1 Internal Medicine Department, University Medical Center, Street Syganak, 46, 010000 Astana, Republic of Kazakhstan; 2 Department of General Medical Practice, Asfendiyarov Kazakh National Medical University, #1, Street Tole Bi, 94, 050000 Almaty, Republic of Kazakhstan; 3 Department of Endocrinology, Astana Medical University, Street Beibitshilik St 49/A, Astana, Republic of Kazakhstan; 4 Faculty of Postgraduate Education, Asfendiyarov Kazakh National Medical University, Street Tole Bi, 94, 050000 Almaty, Republic of Kazakhstan; 5 Faculty of Internal Medicine, Astana Medical University, Street Beibitshilik St 49/A, Astana, Republic of Kazakhstan; 6 Department of Transfusion Medicine, Semmelweis University, Vas U. 17, Budapest, 1088 Hungary; 7 Faculty of Pathology and Forensic Medicine, Astana Medical University, Astana, Republic of Kazakhstan; 8 ANADETO Medical Center, St. Kerey, Zhanibek Khans, 22, 010000 Astana, Republic of Kazakhstan

**Keywords:** COVID-19, Type 2 diabetes mellitus, Hypertension, NASH, Restricted diet, Fast weight loss, Inflammation/glycemic/lipid profile, Chest CT, Lipid/protein oxidation, Cardiovascular diseases, Endocrine system and metabolic diseases, Infectious diseases, Metabolic disorders, Non-alcoholic steatohepatitis

## Abstract

COVID-19 comorbid with noncommunicable chronic diseases (NCDs) complicates the diagnosis, treatment, and prognosis, and increases the mortality rate. The aim is to evaluate the effects of a restricted diet on clinical/laboratory inflammation and metabolic profile, reactive oxygen species (ROS), and body composition in patients with COVID-19 comorbid with NCDs. We conducted a 6-week open, pilot prospective controlled clinical trial. The study included 70 adult patients with COVID-19 comorbid with type 2 diabetes (T2D), hypertension, or nonalcoholic steatohepatitis (NASH). Interventions: a restricted diet including calorie restriction, hot water drinking, walking, and sexual self-restraint. Primary endpoints: COVID-19 diagnosis by detecting SARS-CoV-2 genome by RT-PCR; weight loss in Main group; body temperature; C-reactive protein. Secondary endpoints: the number of white blood cells; erythrocyte sedimentation rate; adverse effects during treatment; fasting blood glucose, glycosylated hemoglobin A1c (HbA1c), systolic/diastolic blood pressure (BP); blood lipids; ALT/AST, chest CT-scan. In Main group, patients with overweight lost weight from baseline (− 12.4%; *P* < 0.0001); 2.9% in Main group and 7.2% in Controls were positive for COVID-19 (RR: 0.41, CI: 0.04–4.31; *P* = 0.22) on the 14th day of treatment. Body temperature and C-reactive protein decreased significantly in Main group compared to Controls on day 14th of treatment (*P* < 0.025). Systolic/diastolic BP normalized (*P* < 0.025), glucose/lipids metabolism (*P* < 0.025); ALT/AST normalized (*P* < 0.025), platelets increased from baseline (*P* < 0.025), chest CT (*P* < 0.025) in Main group at 14 day of treatment. The previous antidiabetic, antihypertensive, anti-inflammatory, hepatoprotective, and other symptomatic medications were adequately decreased to completely stop during the weight loss treatment. Thus, the fast weight loss treatment may be beneficial for the COVID-19 patients with comorbid T2D, hypertension, and NASH over traditional medical treatment because, it improved clinical and laboratory/instrumental data on inflammation; glucose/lipid metabolism, systolic/diastolic BPs, and NASH biochemical outcomes, reactive oxygen species; and allowed patients to stop taking medications.

Trial Registration: ClinicalTrials.gov NCT05635539 (02/12/2022): https://clinicaltrials.gov/ct2/show/NCT05635539?term=NCT05635539&draw=2&rank=1.

## Introduction

SARS-CoV-2, a virus that causes the disease known as COVID-19, was first described as a case of pneumonia of unknown origin in Wuhan city, China but quickly evolved into a worldwide pandemic, and has changed the mortality rate^1–3^. COVID-19 is the most common acute respiratory disease (ARD) in the world and can quickly spread through the air and direct contact^2,4,5^. ARD is a serious illness that affects > 100 million people worldwide each year; it results in millions of deaths^6,7^. The increased incidence of COVID-19 as a leading cause of death in some age groups is consistent with a downward age shift in the distribution of COVID-19 deaths in recent years^3,8^. 

Noncommunicable chronic diseases (NCDs) are also the leading cause of mortality/morbidity worldwide^9,10^. COVID-19 comorbid with NCDs complicates the diagnosis/treatment/prognosis of the ARD emerging new concern^11,12^. This comorbidity can exacerbate disease symptoms and mortality. The aggravating effects of ARD on cardiovascular comorbidities, diabetes onset, and chronic liver and kidney diseases have long been debated^13,14^. Older age, type 2 diabetes mellitus (T2D), hypertension, and liver and kidney diseases were significantly associated with an increased likelihood of mortality^15^.

Pharmacologic and biologic treatment of ARD and NCDs has yielded positive results. However, the results are insufficient and could be worsened by side effects^9,16,17^. Furthermore, the treatment of ARD is more difficult in patients with comorbidities, such as T2D, hypertension, nonalcoholic steatohepatitis (NASH), and obesity^18–20^. With the continuous emergence of SARS-CoV-2 variants, COVID-19 is considered to remain significant public concerns in the future^3,21^. Therefore, establishment of reliable, safe, and natural methods for the treatment of COVID, particularly in patients with NCDs, are required^3,5,17,21,22^.

NCDs and COVID-19 are problematic in countries with a higher rate of overweight^12,23–27^. The Comprehensive Assessment of the Long-term Effects of Reducing Intake of Energy (CALERIE) phase 2 trial showed that a moderate reduction in energy intake averaging approximately 12% over 2 years could improve markers of inflammation, cardiometabolic health, and oxidative stress in humans^28,29^. Caloric restriction has been shown to extend the lifespan and health span of many animal species^30^. There has been growing interest in evaluating related strategies, such as intermittent fasting, periodic fasting, and time-restricted eating, that may achieve the putative benefits of caloric restriction with a greater likelihood of sustainability^31^.

COVID-19 affects different people in different ways. Most infected people will develop mild to moderate illness and recover without hospitalization. Some studies reported a high prevalence of overweight and obesity in patients experiencing a severe COVID-19 course, with serious complications requiring hospitalization and admission to intensive care units^27^. Patients with obesity regularly take medications for the treatment of any concurrent chronic diseases, and physicians must promptly manage any medical symptoms in the case of suspected acute respiratory syndrome infection to prevent severe outcomes^32^.

ARD with overweight is associated with an increase in oxidative stress and a decrease in antioxidant protection^23^. Acute inflammation in patients with overweight is extremely difficult to treat, highlighting the need for preventive measures^33^.

Pharmacological treatment of ARD with NASH is a great challenge because patients are often limited from taking medicine due to persistent progression, increased hepatocellular injury/inflammation, and medicinal overloading^19^.

In previous studies, we showed the significant impact of overweight/obesity on the increasing COVID-19 morbidity/mortality^26^, and the beneficial role of a restricted diet in improving glycemic, lipid, and hormone profiles; electrolyte and biochemical indices; blood pressure; reactive oxygen species; and bone mineral density in patients with T2D/hypertension/severe NASH^34,35^. The aim of this study was to evaluate the effects of a restricted diet on clinical and laboratory inflammation, metabolic profile, reactive oxygen species (ROS), and body composition in patients with COVID-19 comorbid with T2D/hypertension/NASH.

## Study design and participants

### Study design

A 6-week, open, pilot prospective controlled clinical trial with the intention-to-treat principle.

### Participants

**Figure 1:**
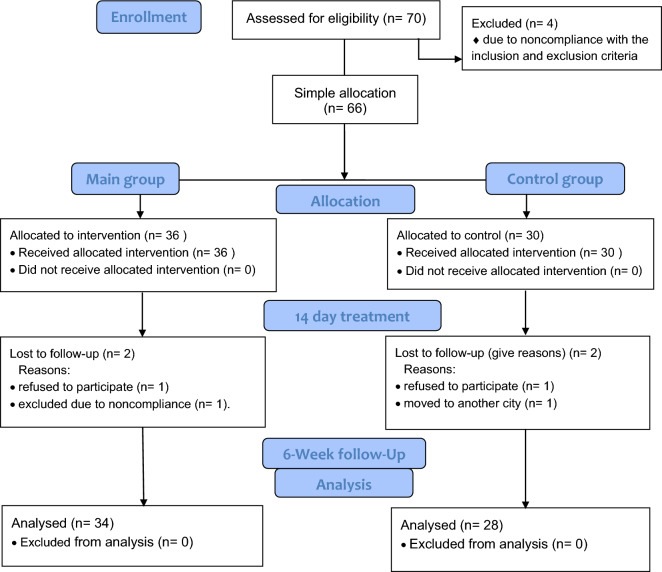
CONSORT 2010 flow diagram. Weight loss treatment of COVID-19 in patients with NCDs: a pilot prospective clinical trial.

In total, 70 adults (43 women) aged 25–80 years with moderate-to-severe cases of COVID-19 comorbid with T2D, hypertension, or NASH were enrolled; four patients were excluded due to noncompliance with the inclusion and exclusion criteria as explained below. In the ITT analysis were included 66 patients (40 women, 39 patients ≥ 50 years old, 58 Asian ethnicity). The patients were simply non-randomly divided into two groups: the experimental group (Main group), 36 patients; and the control group (Controls), 30 patients (Fig. 1). The non-random allocation was performed to divide patients to the two groups based on both patient and researcher choices. The main reason for inclusion of patients in Main group was that the majority of patients refused pharmacology therapy either due to previous unsuccessful drug results, antimicrobial resistance profile, drug allergies, or because of the risk of developing severe symptoms of NASH. Main group received a restricted diet, and Controls received conventional antiviral, immunomodulatory, pathogenetic, and symptomatic pharmacologic therapy^36,37^.

Four of the remaining 66 patients (6.1%) dropped out before study completion: two refused the treatment methods for 3 days after starting, one moved to another city, and one was excluded due to noncompliance. Thus, 62 patients (38 women, 37 patients ≥ 50 years old, 57 Asian ethnicity) were included for the final analysis: 34 patients in Main group; and 28 patients in Controls.

In Main group overweight in 21 patients (61.7%) with body mass index (BMI) 29.10 ± 0.38 kg/m^2^, and 13 without overweight with BMI 23.87 ± 0.38 kg/m^2^. In Controls overweight in 16 patients (57.1%) with body mass index (BMI) 28.07 ± 0.41 kg/m^2^, and 12 without overweight with BMI 23.73 ± 0.36 kg/m^2^. In Main group T2D in 18 (52.9%); Hypertension in 21 (61.8%) (incl. 12 with T2D); NASH in 28 (82.3%) (incl. 8 with NASH/T2D/Hypertension; 7 with NASH/T2D; 13 with NASH/Hypertension). In Controls T2D in 12 (42.8%); Hypertension in 18 (64.3%) (incl. 7 with T2D); NASH in 17 (60.7%) (incl. 5 with NASH/T2D/Hypertension; 3 with NASH/T2D; 9 with NASH/Hypertension). Main group patients in compare with Controls were clinically more severe subjectively, but it was not significantly (Tables 1 and 2)^38,39^.

**Table 1:** Anthropometric data, body composition of patients (with/without overweight) in Main group (n = 34) and Controls (n = 28) at baseline, during treatment days, and at the 6-week follow-up (M ± SEM).

Variables	Main group				Controls			
	Baseline	Treatment days		6-Week follow-up	Baseline	Treatment days		6-Week follow-up
		7th day	14th day			7th day	14th day	
Weight in patients with overweight, kg (21 in Main group, 16 in Controls)	84.11 ± 0.74	76.16 ± 0.78*	72.13 ± 0.77*^#^	73.22 ± 0.77*^#^	82.07 ± 0.71	81.9 ± 0.70	80.9 ± 0.70	82.12 ± 0.73
BMI (kg/m^2^)	29.10 ± 0.38	26.35 ± 0.37*^#^	24.96 ± 0.36*^#^	25.34 ± 0.36*^#^	28.07 ± 0.41	27.98 ± 0.40	27.83 ± 0.39	28.24 ± 0.39
Fat mass (%)	29.88 ± 0.46	22.81 ± 0.44*	21.63 ± 0.48*	22.38 ± 0.44*	–	–	–	–
Fat-free mass (kg)	57.87 ± 0.45	57.56 ± 0.44	57.03 ± 0.43	57.12 ± 0.42	–	–	–	–
Total body water (%)	53.12 ± 0.44	57.39 ± 0.44*	63.12 ± 0.44*	59.46 ± 0.44*	–	–	–	–
Muscle mass (%)	65.33 ± 0.98	68.24 ± 1.01	70.41 ± 0.99*	70.02 ± 0.98*	–	–	–	–
Bone mass (%)	3.62 ± 0.09	3.68 ± 0.1	3.70 ± 0.09	3.69 ± 0.10	–	–	–	–
Weight in patients without overweight, kg (13 in Main group, 12 in Controls)	68.97 ± 0.62	64.21 ± 0.63*^#^	62.64 ± 0.61*^#^	63.16 ± 0.61*^#^	69.39 ± 0.74	68,73 ± 0.72	67.86 ± 0.72	68.27 ± 0.72
BMI (kg/m^2^)	23.87 ± 0.38	22.22 ± 0.37*^#^	21.67 ± 0.37*^#^	21.85 ± 0.36*^#^	23.73 ± 0.36	23.48 ± 0.36	23.21 ± 0.36	23.34 ± 0.36
Fat mass (%)	24.58 ± 0.41	23.41 ± 0.41	22.73 ± 0.42*	22.06 ± 0.42*	–	–	–	–
Fat-free mass (kg)	51.92 ± 0.52	52.04 ± 0.51	52.44 ± 0.52	52.15 ± 0.53	–	–	–	–
Total body water (%)	53.78 ± 0.46	55.23 ± 0.46	59.92 ± 0.45*	56.39 ± 0.45*	–	–	–	–
Muscle mass (%)	66.87 ± 0.97	68.55 ± 0.99	69.95 ± 0.99	69.17 ± 0.98	–	–	–	–
Bone mass (%)	3.75 ± 0.08	3.80 ± 0.09	3.81 ± 0.10	3.80 ± 0.09	–	–	–	–

*P-value of < 0.025 was considered as significant from baseline in the same group.

^#^*P*-value of < 0.05 was considered as significant difference in Main group from Controls in the corresponding parameters.

*BMI* body mass index, *M* mean, *SEM* standard error of the mean.

**Table 2:** Body temperature, blood pressures, inflammation level, glucose and lipids metabolism, lipid and protein oxidative products, and chest CT in Main group (n = 34) and Controls (n = 28) at baseline, during treatment days, and at the 6-week follow-up (M ± SEM).

Variables (n)	Main group				Controls			
	Baseline	Treatment days		6-Week follow-up	Baseline	Treatment days		6-Week follow-up
		7th day	14th day			7th day	14th day	
Body temperature, °C (34 in Main group, 28 in Controls)	40.03 ± 0.06	37.14 ± 0.08*^#^	36.71 ± 0.05*^#^	36.45 ± 0.03*^#^	39.86 ± 0.06	38.72 ± 0.06*	37.12 ± 0.06*	36.58 ± 0.05*
Systolic BP in patients with hypertension, mmHg (21 in Main group, 18 in Controls)	161.9 ± 1.12	121.5 ± 1.12*^#^	119.7 ± 0.53*^#^	120.8 ± 1.17*^#^	160.2 ± 1.31	153.6 ± 1.26*	143.3 ± 1.23*	131.6 ± 1.17*
Diastolic BP in patients with hypertension, mmHg (21 in Main group, 18 in Controls)	101.8 ± 0.83	77.86 ± 0.75*^#^	81.24 ± 0.67*^#^	80.93 ± 0.67*^#^	99.2 ± 0.88	97.52 ± 0.76*	91.18 ± 0.77*	89.45 ± 0.79*
Total protein, g/L (34 in Main group, 28 in Controls)	61.05 ± 0.59	–	66.87 ± 0.57*^#^	70.44 ± 0.62*^#^	60.89 ± 0.57	–	63.11 ± 0.58*	64.82 ± 0.61*
Hemoglobin, g/L (34 in Main group, 28 in Controls)	127.8 ± 0.70	–	132.1 ± 0.66*^#^	138.1 ± 0.69*^#^	128.5 ± 0.70	–	128.4 ± 0.72	129.2 ± 0.69
White blood cells, × 10^9^/L (34 in Main group, 28 in Controls)	12.27 ± 0.15	10.71 ± 0.16*^#^	7.76 ± 0.12*^#^	5.23 ± 0.09*^#^	12.86 ± 0.15	12.43 ± 0.16	11.53 ± 0.14*	8.11 ± 0.12*
Neutrophil, × 10^9^/L (34 in Main group, 28 in Controls)	7.214 ± 0.012	6.423 ± 0.011*^#^	3.647 ± 0.009*^#^	2.510 ± 0.012*^#^	7.352 ± 0.012	7.147 ± 0.011*	6.492 ± 0.009*	4.967 ± 0.011*
Lymphocytes, × 10^9^/L (34 in Main group, 28 in Controls)	2.512 ± 0.009	3.215 ± 0.009*^#^	2.949 ± 0.009*^#^	1.831 ± 0.008*^#^	2.641 ± 0.009	2.351 ± 0.009*	2.978 ± 0.009*	2.190 ± 0.008*
The neutrophil-to-lymphocyte ratio (34 in Main group, 28 in Controls)	2.872 ± 0.009	1.998 ± 0.008*^#^	1.237 ± 0.009*^#^	1.371 ± 0.008*^#^	2.784 ± 0.008	3.040 ± 0.008*	2.180 ± 0.009*	2.268 ± 0.008*
Erythrocyte sedimentation rate, mm/hour (34 in Main group, 28 in Controls)	35.2 ± 0.52	16.22 ± 0.48*^#^	13.89 ± 0.22*^#^	7.43 ± 0.23*^#^	36.1 ± 0.53	28.61 ± 0.49*	21.94 ± 0.24*	14.62 ± 0.24*
Total Fibrinogen, g/L (34 in Main group, 28 in Controls)	4.91 ± 0.08	5.16 ± 0.08*^#^	3.67 ± 0.07*	2.89 ± 0.08*^#^	4.88 ± 0.07	4.32 ± 0.08*	3.81 ± 0.07*	3.51 ± 0.08*
C-reactive protein, mg/L (34 in Main group, 28 in Controls)	34.44 ± 1.23	18.23 ± 1.19*^#^	11.94 ± 0.72*^#^	6.61 ± 0.45*^#^	32.19 ± 1.23	28.61 ± 1.14	20.43 ± 1.13*	11.58 ± 0.99*
Glucose in patients with T2D, mmol/L (18 in Main group, 12 in Controls)	13.19 ± 0.26	6.86 ± 0.12*^#^	4.89 ± 0.08*^#^	4.81 ± 0.08*^#^	12.97 ± 0.27	9.95 ± 0.13*	7.84 ± 0.12*	6.41 ± 0.09*
HbA1c, % (18 in Main group, 12 in Controls)	7.09 ± 0.09	–	–	4.79 ± 0.08*^#^	7.09 ± 0.09	–	–	6.86 ± 0.09*
Immunoassay Insulin, nU/L (18 in Main group, 12 in Controls)	23,17 ± 0.52	–	9.63 ± 0.23*^#^	6.41 ± 0.12*^#^	22.43 ± 0.49	–	20.56 ± 0.41*	21.49 ± 0.37
HOMA-IR index (18 in Main group, 12 in Controls)	13.58 ± 0.34	–	2.09 ± 0.18*^#^	1.37 ± 0.17*^#^	12.93 ± 0.33	–	7.16 ± 0.25*	6.12 ± 0.22*
Platelets, × 10^9^/L (28 in Main group, 17 in Controls)	185,9 ± 3.56	197.5 ± 4.02	238.5 ± 5.8*^#^	302.8 ± 6.9*^#^	193.9 ± 4.43	196.7 ± 4.94	208.6 ± 5.45	221.6 ± 6.12*
ALT, U/L (28 in Main group, 17 in Controls)	136.4 ± 4.0	104.1 ± 4.3*^#^	77.8 ± 4.4*^#^	26.7 ± 1.9*^#^	134.7 ± 4.2	119.4 ± 3.9*	97.3 ± 3.8*	67.3 ± 3.1*
AST, U/L (28 in Main group, 17 in Controls)	169.4 ± 4.2	123.7 ± 4.1*^#^	90.8 ± 4.8*^#^	29.5 ± 1.8*^#^	158.7 ± 4.3*	137.9 ± 4.1*	76.8 ± 3.8*	59.7 ± 3.2*
Urea, mmol/L (34 in Main group, 28 in Controls)	6.14 ± 0.08	7.32 ± 0.09*	5.51 ± 0.06*^#^	4.19 ± 0.07*^#^	6.07 ± 0.08	5.94 ± 0.07	5.62 ± 0.06*	4.85 ± 0.07*
Creatinine, µmol/L (34 in Main group, 28 in Controls)	76.29 ± 1.12	108.78 ± 1.48*^#^	71.14 ± 1.07*	59.21 ± 0.93*^#^	74.66 ± 1.17	71.91 ± 1.07	70.86 ± 1.02*	68.45 ± 0.94*
Cholesterol, mmol/L (34 in Main group, 28 in Controls)	5.97 ± 0.08	6.86 ± 0.08*^#^	6.18 ± 0.07^#^	4.77 ± 0.05*^#^	6.01 ± 0.08	5.94 ± 0.08	5.83 ± 0.07	5.29 ± 0.06*
Triglyceride, mmol/L (34 in Main group, 28 in Controls)	2.48 ± 0.06	5.89 ± 0.07*^#^	5.74 ± 0.08*^#^	1.13 ± 0.05*^#^	2.54 ± 0.06	2.63 ± 0.07	2.24 ± 0.06*	1.97 ± 0.05*
HDL, mmol/L (34 in Main group, 28 in Controls)	0.95 ± 0.03	1.03 ± 0.03^#^	1.06 ± 0.03*	1.69 ± 0.04*^#^	0.95 ± 0.03	0.93 ± 0.03	0.97 ± 0.03	1.01 ± 0.03*
MDA, µmol/L (34 in Main group, 28 in Controls)	64.61 ± 1.72	–	40.57 ± 0.92*^#^	32.43 ± 0.90*^#^	69.63 ± 1.84	–	59.32 ± 1.47*	52.72 ± 1.21*
AOPP, µmol/L (34 in Main group, 28 in Controls)	298.8 ± 7.9	–	208.7 ± 8.7*^#^	178.4 ± 7.4*^#^	279.4 ± 9.2	–	267.3 ± 8.9	237.8 ± 7.9*
SOD, U/mg (34 in Main group, 28 in Controls)	32.54 ± 1.01	–	62.72 ± 1.06*^#^	71.41 ± 1.03*^#^	35.23 ± 1.07	–	41.28 ± 1.06*	47.36 ± 1.05*
Catalase, U/g (34 in Main group, 28 in Controls)	23.21 ± 0.54	–	44.99 ± 0.67*^#^	51.02 ± 0.66*^#^	25.01 ± 0.67	–	27.38 ± 0.65	34.29 ± 0.71*
Chest CT scan, score (34 in Main group, 28 in Controls)	13.09 ± 0.29	–	1.74 ± 0.13*^#^	–	12.86 ± 0.31	–	4.28 ± 0.15*	–
Positive COVID-19 (34 in Main group, 28 in Controls)	34	–	1 (2.9%)	0 (0%)	28	–	2 (7.2%)	1 (3.6%)

*P-value of < 0.025 was considered as significant from baseline in the same group.

^#^*P*-value of < 0.05 was considered as significant differences in Main group from Controls in the corresponding parameters.

*AOPP* advanced oxidation protein products, *BP* blood pressure, *CT* computed tomography, *HDL* high-density lipoprotein, *HOMA-IR* Homeostasis Model Assessment for Insulin Resistance, *M* mean, *MDA* malondialdehyde, *SOD* superoxide dismutase, *SEM* standard error of the mean, *T2D* type 2 diabetes mellitus.

#### Justification of the sample size

The estimated treatment difference was set to 10% with a standard deviation of 8%, margin on risk difference scale of 0.35, and superiority margin of 5% (δ = 0.05)^40^ based on two-sided hypothesis testing. Using SPSS, Sample-Power, V23.0, the number of evaluable individuals needed per treatment arm was > 27. At least 54 patients will be screened and recruited for this comparative clinical trial^41^. We recruited patients until the study population reached > 60 patients and Main group > 30.

All patients with ARD comorbid with one or more NCDs. All patients with T2D were treated with metformin, sulfonylurea, DPP-4 inhibitors, GLP-1 receptor agonists, and glitazones in various combinations, but at least two drugs per patient. Patients with hypertension received antihypertensive standard treatment (calcium channel blockers, angiotensin converting enzyme inhibitors, angiotensin receptor blockers, diuretics, beta-blockers, vasodilators, and centrally acting agents in various combinations but at least two drugs per patient). The data of Controls were taken from the hospital inpatient department of Astana Municipal Clinic #2. The data of Main group were taken from the Republican Diagnostic Center at University Medical Center and ANADETO Medical Center (Astana).

In both groups, the majority of patients wanted to stop taking medications either because of previous unsuccessful drug results, antimicrobial resistance profiles, or drug allergies; or a history of severe NASH symptoms. Main group started with medication + weight loss and Controls started with medication only. In Main group there was no intention to stop taking the medications.

#### Inclusion criteria

(1) Written informed consent; (2) 25 ≤ age < 80 years old; (3) patients with fever (> 38.5 °C); (4) patients with moderate-to-severe cases of COVID-19 (according to having oxygen saturation > 93% by finger oximetry at resting status; lung infiltrates by a chest computed tomography (CT) radiographic score > 13 at max of 24, i.e. > 50% lung damage; > 60% of patients aged ≥ 50 years old; > 60% of patients have NASH; high levels of BP, C-reactive protein, and ALT/AST) diagnosed within 1–2 days and not receiving treatment, as well as with comorbid NCDs; (5) the availability of CT scan data obtained within 1–2 days of COVID-19 diagnosis (baseline) and on day 14 of treatment; 6) treatment for 14 days and + 4 weeks follow-up (total 6 weeks).

#### Exclusion criteria

(1) Age < 25 or ≥ 80 years old; (2) acute respiratory failure and assisted ventilation requirement; (3) respiratory rate ≥ 30 times/minute; (4) oxygen saturation ≤ 93% by finger oximetry at resting status; and (5) malignancy within the past 5 years; gestation or lactation; kidney failure; hereditary diseases; or known hypersensitivity to any of the test substances.

#### Outcome measures

*Primary endpoints:* COVID-19 diagnosis by detecting SARS-CoV-2 genome by RT-PCR; weight loss for 14 days in Main group; body temperature; and C-reactive protein. *Secondary endpoints:* white blood cells; erythrocyte sedimentation rate; adverse effects during treatment; glycosylated hemoglobin A1c (HbA1c); systolic/diastolic blood pressure (BP); liver test results (alanine aminotransferase, ALT; aspartate aminotransferase, AST; platelet); metabolic test (lipids, glucose metabolism, hemoglobin, total protein); and CT scan.

#### Intervention

In Main group we used a restricted diet for weight loss (“Analimentary detoxication”, ANADETO) including calorie restriction to 50–100 kcal/day with fat-free vegetables (tomatoes and cucumbers) with mandatory salt (NaCl) intake to 5–6 g/day based on WHO’s recommendation (2019), hot water drinking 1000–1500 mL/day, walking at least 2000 steps/day after body temperature normalization, and sexual self-restraint^42^. The restricted diet provided a very-low-calorie diet and fast weight loss. Walking maintains body structure; improves blood circulation, blood perfusion through detoxification organs (skin, kidney, liver, digestive tract, and lungs) that clearing the blood from metabolic substances that appear during weight loss; increases oxygen saturation^43–48^. The weight loss lasted 14 days, after which the patients followed a 4-week diet with one meal a day (in the evening) without any food restriction. A combination of in-person conversations and telephone calls was conducted during the whole 6-week study period.

Standard medical therapy in Controls included conventional antiviral, immunomodulating, nonsteroidal and steroidal anti-inflammatory drugs, and other pathogenetic symptomatic pharmacologic therapies according to the following indications [https://online.zakon.kz/Document/?doc_id=36043894&pos=6;-106#pos=6;-106, The clinical protocol for diagnosis and treatment coronavirus infection COVID-19 in adults, in Kazakhstan]^36,37^. In both groups, treatment started immediately within one or two days of a positive COVID-19 result.

### Analytical assessment

#### Pathogen detection methods

##### Infection detection

COVID-19 was diagnosed by direct detection of SARS-CoV-2 RNA via nucleic acid amplification tests with a real-time reverse-transcriptase polymerase-chain-reaction (RT-PCR) assay using nasal or pharyngeal swab specimens from the upper respiratory tract. A healthcare professional collected a fluid sample by inserting a long nasopharyngeal swab into a nostril and taking fluid from the back of the nose.

Sputum specimen collection, transport, storage, diagnostic test procedure environment, and test result reporting were performed strictly following the standard guidelines^49,50^.

All the tests were performed in two independent laboratories in Astana: the BioLab and OLIMP laboratories (https://bio-lab.kz/ and https://www.kdlolymp.kz/). Positive results were considered when both laboratories provided the same results.

*Biochemical tests* included of a complete blood cell counts, white blood cells (normal: 3.5–9.0 × 10^9^/L), neutrophil-to-lymphocyte ratios (NLR) calculated by dividing the absolute count for neutrophils by the absolute count for lymphocytes (normal: 1–2), blood chemical analysis, erythrocyte sedimentation rate, urea (normal: 2.1–7.7 mmol/L), creatinine (normal: 53–115 µmol/L), glucose (normal: 3.7–5.8 mmol/L), cholesterol (normal: < 5.4 mmol/L), triglyceride (normal: < 1.69 mmol/L), high-density lipoprotein (HDL) (normal: > 1.55 mmol/L), fibrinogen (normal: 2.0–4.0 g/L), hepatic enzymes (ALT, norm < 36 U/L, AST, norm < 40 U/L), liver and renal function, inflammation level, C-reactive protein (normal: ≤ 10.0 mg/L), and HbA1c (normal: < 5.7%; prediabetes: 5.7–6.5%, diabetes: ≥ 6.5%). Demographic data, clinical symptoms, clinical outcomes, and laboratory test results were included in the electronic medical records.

##### Hormonal assays

Fasting serum insulin was determined via an immunoassay (Immunotech Insulin Irma Kit, Prague, Czech Republic). Hyperinsulinemia was considered > 12.5 nU/L. The Homeostasis Model Assessment insulin resistance index (HOMA-IR) was used as a surrogate measure of insulin sensitivity as follows: HOMA-IR = ((fasting insulin in nU/L) × (fasting glucose in mmol/L)/22.5); insulin resistance was considered if the index was > 2. The Patients temporarily stopped taking antidiabetic medications 24 h before blood collection to determine insulin.

The standard of the American Diabetes Association (2017) was used for T2D diagnosis^51^. Hypertension was diagnosed by BP readings and from medical records. The physical activity of patients was assessed by the number of steps measured by pedometers (Hoffmann-La Roche Ltd, Basel, Switzerland).

The Nonalcoholic Steatohepatitis Clinical Research Network criteria were used for NASH diagnosis^52^.

##### Anthropometric data

Anthropometric indices included age (years), weight (kg), and BMI (kg/m^2^). Body composition parameters including fat mass (in % of total body weight and total kg), fat-free mass (kg), total body water (%), muscle and bone mass (%) were measured using Tanita-SC330S Body Composition Analyzer (Tanita Corp., Japan). We measured body composition only in Main group, who lost weight.

*For ROS* we measured malondialdehyde (MDA)^53^, superoxide dismutase (SOD)^54^ and catalase (antioxidant enzymes)^55^, and advanced oxidation protein products (AOPP). MDA was measured by using thiobarbituric acid-reactive substance assay as described by Yagi with modification of Ohkawa^53^. Antioxidant enzymes were determined in erythrocyte hemolysates, in which hemoglobin concentration was assayed using Drabkin's method. SOD was measured by the method of Misra and Fridrovich^54^. Catalase was measured at λ = 240 nm at 25 °C. Catalase activity unit (U) was taken to be the amount of the enzyme which decomposes 1 g of H_2_O_2_ in 1 min at 25 °C in pH = 7.0. AOPP were measured by adding 40 µL of plasma 190 µL of mixture of 81% phosphate buffer solution, 10% glacial acetic acid and 4% 1.16 mmol solution of KI, then for 2 min absorbance at 340 nm on plate reader Multiscan Ascent using spectrophotometric method. As the calibrator, chloramine-T solution was used^55^.

##### Adverse effects during treatment (AEs)

The symptoms of AEs were screened by the patient’s claims/complaints, such as headache, abdominal pain, vomiting/nausea/diarrhea, hypoglycemia, hard-to-control BP, increased hepatic enzymes, and any allergic reaction in both compared groups during the 14-day treatment and 6-week follow-up.

#### Imaging

Ultrasound images (GE-Vivid-9-Ultrasound; GE-Healthcare-Worldwide-USA, Michigan), and chest CT (Siemens-Somatom-Sensation-32) were obtained. The key imaging findings in the pneumonia was ground-glass opacities, distribution, and extensive of lung abnormalities, including consolidation, cavitation, and pleural effusion.

Chest CT assessment of the severity of lung changes. Each lung field was divided into three equal zones. Each zone was assigned a score from 0–4 based on the percentage of lungs involved (0 = no abnormality, 1 = < 25% of the zone involved, 2 = 25–50% involved, 3 = 51–75% involved, and 4 =  > 75% involved). The scores for all six zones of each CT examination were summed to provide a cumulative chest radiographic score (range, 0–24); CT severity was graded as low (0–3), mild (4–7), moderate (8–12), and severe (> 13)^56,57^. Baseline and follow-up CT scans were reviewed in consensus by two radiologists with 10–25 years of experience. Laboratory tests, ultrasound/CT images, and an electrocardiogram were performed, and sputum and blood samples were collected on the inclusion day.

### Statistics

Two-sided Student’s *t* tests with Bonferroni correction and the Chi-square tests were used for categorical variables, as appropriate. *P* value of < 0.025 was set as significant differences in intragroup and < 0.05 between groups. The study data were tested for a normal distribution and are presented in tables as Mean ± Standard Error of the Mean (M ± SEM). Statistical analysis was performed using SPSS ver.23.0 for Windows (SPSS: An IBM Company, Armunk, NY) and Microsoft Excel-2021. All analyses were intention-to-treat.

### Ethics approval and consent to participate

The study protocol was approved (approval protocol number is #7 of 26.09.2019) by the Local Ethics Committee of the University Medical Center (phone: + 71272–692586; e-mails: mirgul.bayanova@umc.org.kz; https://umc.org.kz/en/?ethics-commission). The committee confirms that all methods were performed in accordance with the Declaration of Helsinki and guidelines of the Council for International Organizations of Medical Sciences (CIOMS) and that informed consent was obtained from all participants.

### Declaration

The study was carried out in the Republic of Kazakhstan from November 2020, through July 2022. Participants were recruited gradually as they arrived at the Republican Diagnostic Center at University Medical Center (Astana) and ANADETO Medical Center from November 2020 to March 2022. 

## Results

In Main group, 21 patients (61.7%) were overweight with BMI 29.10 ± 0.38 kg/m^2^, and 13 were not overweight with BMI 23.87 ± 0.38 kg/m^2^. In Controls, 16 patients were overweight (57.1%) with BMI 28.07 ± 0.41 kg/m^2^, and 12 were not overweight with BMI 23.73 ± 0.36 kg/m^2^. In Main group, 18 (52.9%) had T2D; 21 (61.8%) had hypertension (incl. 12 with hypertension/T2D); 28 had NASH (82.3%) (incl. 8 with NASH/T2D/hypertension; 7 with NASH/T2D; 13 with NASH/Hypertension). Among Controls, 12 had T2D (42.8%); 18 had hypertension (64.3%) (incl. 7 with hypertension/T2D); 17 had NASH (60.7%) (incl. 5 with NASH/T2D/hypertension; 3 with NASH/T2D; 9 with NASH/hypertension).

Symptoms of COVID-19 were common in two groups and included fever in all 62 patients (100%), cough in 48 (77.4%), runny or stuffy nose in 51 (82.2%), fatigue in 49 (79%), chills in 30 (48.4%), sore throat in 14 (22.6%), headache in 22 (35.4%), and body aches in 18 (29%). Loss of taste or smell was observed in 21 patients (33.8%). Loss of appetite and smell irritability were in 51 patients (82.3%).

At baseline, no differences were detected in the main background characteristics between the two groups (Tables 1 and 2).

The baseline/7-day/14-day treatment and 6-week follow-up results concerning the anthropometrical, body composition, and metabolic data are shown in Table 1. There was a trend toward a decrease in weight in Controls (with and without overweight), but the difference was not significant. The weight in Main group decreased in different ways depending on the presence of overweight. In Main group, patients with overweight lost 8–11 kg (− 12.4% from baseline), and it was significantly higher than patients without overweight (− 9.2% from baseline; − 11.9 ± 0.7 kg vs. − 6.3 ± 0.6 kg, respectively; *P* < 0.0001) on the 14th day of the treatment. The decrease in BMI at 14 day from the baseline was also greater in patients with overweight (− 4.2 kg/m^2^) than in patients without overweight (− 2.3 kg/m^2^) (*P* < 0.001).

Changes in clinical and laboratory data in Main group during the treatment were similar in patients with and without overweight.

Weight loss in Main group was due to a reduction in fat mass (*P* < 0.0001) (Table 1). The percentages of total body water and muscle mass tended to increase significantly at 7-day/14-day of treatment, and the percentage of bone mass increased significantly (in patients with overweight *P* < 0.0001; without overweight *P* < 0.05). Lean body mass (fat-free mass) did not change significantly during weight loss in either group of patients, patients with and without overweight (*P* = 0.82–0.97). These trends persisted at 6 weeks of follow-up.

In Main group, after 2–3 days of weight loss treatment, sputum production increased to 1.0–1.5 L/day in all patients, which decreased after 7–9 days of treatment.

In Main group, body temperature decreased from 3–4 days of treatment and normalized to 14 days (*P* < 0.05); however, in Controls, body temperature was still higher at 14 days of treatment (Table 2). In Main group, beginning at 3–5 days of treatment, in most patients, their urine became turbid, muddy, and intensely dark, which persisted for several days. Urine microscopy revealed organic and nonorganic salts such as oxalates/urates/phosphates/carbonates of calcium/magnesium, and leukocyturia (20–35 per in sight). Starting at 5–7 days, the patients noticed physical relief, an increase in physical/mental workability, and exercise tolerance.

COVID-19 stayed positive in one patient in Main group (2.9%) and in two in Controls (7.2%) on the 14th day of treatment (RR: 0.41, CI: 0.04–4.31; *P* = 0.22); and no patients in Main group (0%) and one in Controls (3.6%) at the 6-week follow-up (RR: 0.28; CI: 0.05; 1.61; *P* = 0.13) (Table 2).

The weight loss treatment in Main group evoked a significant increase in serum urea and creatinine levels within 7 days. Normalization of serum urea and creatinine was observed at 14 day of treatment (Table 2).

During weight loss in Main group, total serum protein level significantly increased from baseline (61.05 ± 0.59 g/L) to 14 days of treatment (66.87 ± 0.57 g/L, *P* < 0.0001), and at the 6-week follow-up (70.44 ± 0.62 g/L, *P* < 0.0001). Hemoglobin level also significantly increased from baseline (127.8 ± 0.70 g/L) to 14 days of treatment (132.1 ± 0.66 g/L, *P* < 0.05), and at the 6-week follow-up (138.1 ± 0.69 g/L, *P* < 0.0001). HDL significantly increased from baseline (0.95 ± 0.03 mmol/L) to 14 days of treatment (1.03 ± 0.03 mmol/L,* P* = 0.02), and at the 6-week follow-up (1.69 ± 0.04 mmol/L, *P* < 0.0001) (Table 2). In Controls, serum protein also significantly increased at 14 days of treatment and at the 6-week follow-up, but in Main group compared with Controls the increase was significantly higher (*P* < 0.05).

In Main group, white blood cell counts gradually decreased significantly over the 14 days compared with Controls (*P* < 0.05). In Main group compared with Controls, the relative lymphocyte counts increased, and the NLR was significantly decreased (*P* < 0.05). In Main group, the inflammatory parameters as total fibrinogen, C-reactive protein, and the erythrocyte sedimentation rate significantly decreased to normal levels on the 14th day of treatment (*P* < 0.025) and at the 6-week follow-up (*P* < 0.025), and the improvements in these parameters were greater than in Controls (*P* < 0.05) (Table 2).

In Main group, the weight loss treatment led to a normal systolic/diastolic BP starting from the 7th day of treatment, which was normalized at 14th day of treatment and at the 6-week follow-up, reaching the levels recommended by the American Heart Association (2014), however, in Controls, the BPs were not fully normal at the 14th day and 6-week follow-up (Table 2).

In Main group, the changes in glucose metabolism in patients with T2D (n = 18) were positive at 7 and 14 days of treatment and 6-week follow-up. Fasting glucose and Immunoassay insulin, and HOMA-IR index quickly decreased at 14th day of treatment. Insulin decreased 2.4-fold from baseline at 14 day of treatment but 3.6-fold from baseline at the 6-week follow-up. HbA1c decreased to the normal to the 6-week follow-up (4.79 ± 0.08) by 32% (*P* < 0.05). While in Controls, in patients with T2D, changes in glucose metabolism did not normal on the 14th day of treatment and at 6-week follow-up. Glucose decreased in Controls on 14th day of treatment and at 6-week follow-up, but the level did not reach normal range. Immunoassay insulin, HOMA-IR index, and HbA1c did not reach normal values in Controls.

Lipids increased at the 7 and 14 days during the restricted diet in Main group compared with Controls; lipids decreased at the 6-week follow-up. Lipids increasing in Main group was related to endogenous lipolysis.

In Main group, cholesterol increased in 15–3.5% (*P* < 0.025) at 7 and 14 days, respectively, and triglyceride increased in 2.3-fold from baseline (P < 0.025); however, the levels decreased to normal at the 6-week follow-up. HDL positively increased during all periods of treatment and follow-up (P < 0.025).

In Main group, in patients with NASH (n = 28), ALT/AST levels at all tested days significantly decreased from baseline (*P* < 0.025) to normal at the 6-week follow-up, while in Controls the levels did not reach normal at the 6-week follow-up (Table 2).

In Main group, platelets significantly increased from baseline on the 14th day of treatment (*P* < 0.025), and at the 6-week follow-up (*P* < 0.025), however, in Controls the increase was not significant. In Main group, the increase was significantly greater than in Controls (*P* < 0.05).

In Main group, urea and creatinine significantly increased at 7th day compared to Controls (*P* < 0.05), but these levels decreased at the 14th day of treatment and 6-week follow-up.

In Main group, the oxidative products of lipids/proteins significantly tended to normalize at the 14th day treatment and 6-week follow-up (*P* < 0.025), but in Controls the trend was not substantial. In Main group, MDA decreased in 31.7% at the 14 day (*P* < 0.025) (in Controls only in 15%, *P* < 0.05) and in 48.4% at the 6 week (in Controls in 24.3%, *P* < 0.05) from baseline (*P* < 0.025), AOPP decreased in 30.2% at the 14 day (*P* < 0.025) (in Controls only 5%, *P* < 0.05) and in 36.3% at the 6 week from baseline (*P* < 0.025) (in Controls in 14.8%, *P* < 0.05), and SOD increased in 66.9% (*P* < 0.025) (in Controls only in 17.2%, *P* < 0.05) and Catalase in 76.6% at the 14 day (*P* < 0.025) (in Controls in 9.5%, *P* < 0.05), and in 92.4% and 85.8%, respectively at 6 week from baseline (*P* < 0.025) (in Controls in 34.4% and 37.1%, *P* < 0.05) (Table 2).

### Abnormal chest imaging

CT-scan scores improved more significantly in Main group after 14 days of treatment (from 13.09 to 1.74 in Main group; from 12.86 to 4.28 in Controls; *P* < 0.05) (Table 2). The CT findings included multiple small patchy shadows, centrilobular nodules and bronchial wall thickening, interstitial inflammation, predominantly distributed in periphery of the lungs, ground glass opacities and infiltrates in the lungs, and bronchopneumonia patterns. The most common finding was ground glass opacity (n = 55; 88.7%) not followed by consolidation. In both groups, CT scans taken at the 14th day of treatment showed that the scope of the lesions had decreased, the density was gradually decreased, the number of lesions decreased, and the ground glass opacities were absorbed significantly (*P* < 0.025). Pleural effusion and cavitation were not detected. Unilateral lung involvement (n = 51; 82.2%) was more common than bilateral involvement (n = 11; 17.8%). The right/left lower zones (n = 48; 77.4%) were more commonly affected than the right/left middle (n = 14; 22.6%). Multifocal distribution was more common (n = 35, 56.4%) than unifocal distribution (n = 27, 43.6%).

In Main group, there was no 6-week risk of arterial thromboembolism or venous thromboembolism symptoms, but in Controls was 1 patient (3.6%) who underwent intensive resuscitation.

Both groups at the beginning of the study took antidiabetic, antihypertensive, anti-inflammatory, hepatoprotective, and other symptomatic medications. But in Main group, starting from 2–5 days of the weight loss treatment, it is necessary to reduce and completely stop taking the previous antidiabetic (with T2D, n = 18), antihypertensive (hypertension, n = 21), anti-inflammatory and hepatoprotective (NASH, n = 28), and other symptomatic medications in order to avoid hypoglycemic coma, hypotensive/hypotonic and other effects of drugs. By 5–8 days after treatment started, the drugs were stopped completely in Main group, and there was no T2D, hypertension, or NASH recurrence at the 6-week follow-up. Such a trend was not observed in Controls.

The qualitative and quantitative AEs during treatment in the compared groups are presented in Table 3. The symptoms of AEs were screened by patient claims/complaints in both groups, and symptoms, such as abdominal pain, vomiting, nausea, and diarrhea were observed higher in Controls than in Main group (*P* < 0.05). In Main group, such symptoms as hypoglycemia, hard-to-control BP, increased hepatic enzymes, and allergic reactions were not observed.

**Table 3:** Adverse effects during treatment in Main group (n = 34) and Controls (n = 28) during the 14-day treatment and 6-week follow-up (Chi-square test). Significant values are in bold.

Adverse effects	Main group		Controls		*P*-value
	n	%	n	%	
Headache	14	41.2	19	67.9	0.066
Abdominal pain	5	14.7	12	42.9	**0.029**
Vomiting/nausea/diarrhea	11	32.4	20	71.4	**0.005**
Hypoglycemia	0	0	5	17.9	**0.036**
Hard-to-control BP	0	0	7	25	**0.007**
Hepatic enzymes increasing	0	0	13	46.4	**> 0.0001**
Any allergic reaction	0	0	6	21.4	**0.016**

*BP* blood pressure.

## Discussion

This study demonstrated the beneficial effects of weight loss, based on the restricted diet, for treatment in the COVID-19 patients with unhealthy body conditions, including overweight, T2D, hypertension, and NASH. Overweight patients lost weight faster than non-overweight patients. The difference in weight loss physiology might be explained by differences in fat structure between people with and without overweight^58^. People who quickly gain weight can accumulate lightweight body fat, and people who slowly gain weight can accumulate dense body fat. Some people cannot gain weight due to differences in genotype, fat structure, or metabolic equivalence^59,60^.

Though not reaching statistical significant, this suggested a trend towards a benefit in achieving COVID-19 outcomes: in Main group 2.9% and in Controls 7.2% patients stayed positive at 14th day of treatment; in Main group 0% and in Controls 3.6% patients stayed positive at 6 weeks follow-up.

Weight loss in Main group facilitated normalization of lymphocyte and neutrophil counts, which had been decreased and increased, respectively, due to COVID-19 before treatment. Lymphopenia is also associated with severe illness and poorer survival at COVID-19^61,62^. In clinical practice, the “divergence” between the absolute value of neutrophils and lymphocytes, that NLR, may be correlated with the progression/prognosis of COVID-19^63^.

Our clinical study confirmed that weight loss significantly reduces inflammatory biomarkers, such as total fibrinogen, C-reactive protein, erythrocyte sedimentation rate^64,65^. Reducing inflammation due to weight loss could be associated with a reduced risk of NCDs^28,66^. Weight loss has an immunomodulatory effect, insulin resistance was reversed, blood insulin and BP decreased, and blood hemoglobin increased^67,68^. Blood cholesterol/triglyceride and lipid/protein oxidative products also decreased, and antioxidative enzymes increased.

In patients with NASH, hepatic enzymes ALT and AST in the plasma significantly decreased from baseline to the normal range in Main group, but in Controls the levels of the enzymes were still higher than normal even at the 6-week follow-up, suggesting persisted hepatic damage. This may have been related to the fact that the patients in Controls were treated with antidiabetic, antihypertensive, anti-inflammatory and other hepatotoxic drugs for a longer period of time, while the drug use in Main group was stopped during the weight loss treatment; weight loss has a beneficial effect on liver function in patients with steatohepatitis^35,69,70^.

A great decrease in fat mass by weight loss treatment is considered due to increased lipolysis, as suggested by temporal increases of cholesterol, HDL, and triglyceride observed at 7 and 14 days of treatment in Main group. The parameters were restored to normal ranges by 6-week follow-up. In addition, urea and creatinine also showed temporal increase, which may be caused by active lipolysis or proteolysis. Some humans and animals studies indicated that temporal restriction diets did not induce muscle atrophy or proteolysis^71,72^. We suppose that weight loss in Main group was due to lipolysis because the restriction diet did not reduce lean body mass (fat-free mass) during the weight loss treatment. Weight loss in Main group occurred due to a decrease in fat mass, and the percentage of total body water and muscle mass tended to increase significantly.

Calorie restriction can degrade of endogenous protein-conjugated substrates in different compartments in the body^73,74^. Some studies showed that autophagic proteolysis with lysosomal degradation significantly improved the liver and kidney functions, and prevented aging processes^75–77^. Nutrient deprivation or dietary restriction confers protection against ageing and stress in many animals and induced lysosomal autophagy is part of this mechanism.

An increase in serum urea/creatinine levels during 14-day weight loss in Main group is more indicative of endogenous/metabolic intoxication associated with active lipolysis^78,79^. Adipose tissue is optimized for storing energy in the form of triglycerides, but they also store proteins, vitamins, toxins, drugs, persistent endogenous organic pollutants, and regulatory hormones^80–84^. Adipocytes play specific storing roles in cancer development^85^. Endogenous organic metabolites in adipocytes are eliminated through the blood system during lipolysis^81^.

Previous studies showed that stored endogenous organic metabolites in adipocytes were eliminated during weight loss^46,47,86,87^. An increase in lipids (cholesterol/triglyceride) from baseline at the 7th day of weight loss can confirm the endogenous lipolysis activity. The levels decreased at 14th day of treatment and 6-week follow-up.

One of the requirements for the patients during the weight loss was the mandatory intake of salt (NaCl) in order to eliminate endogenous organic metabolites from the body, as our previous studies have shown^86,87^. Patients with acute respiratory inflammation comorbid with NCDs lose many sodium ions through urine^35,45,88,89^ possibly, therefore in our study blood parameters returned to normal due to the elimination of the metabolic pollutants.

Sodium and chloride are the main transporters of many substrates (nutrients, catabolites) in the body^90,91^. All catabolites excreted from the body contain sodium and/or chloride ions^92^. Salt intake during the weight loss on ANADETO is mandatory for eliminating metabolic excrement from the body.

Many studies suggest that weight loss improves liver health, cardiovascular risk, quality of life, and also improves liver function in patients with NASH^93,94^.

Our study also showed that chest CT findings are highly variable and nonspecific in patients with COVID-19, which has been suggested by other authors^95^. Chest CT has been widely used during the COVID-19-pandemic and has a sensitivity of 60–97%, but low specificity (20–50%)^95,96^. Many professional radiologist recommend against performing chest CT as a primary technique for COVID-19 pneumonia diagnosis^97^. In patients with ARDs, CT findings should be interpreted in combination with the clinical context and direct infection detection by PCR.

In Controls, qualitative and quantitative AEs during treatment were significantly more common in compare with Main group. Symptoms such as hypoglycemia, hard-to-control BP, increased hepatic enzymes, and allergic reactions were not observed in Main group, possibly due to withdrawal of the symptomatic drugs due to necessary to reduce and completely stop taking the previous drugs to avoid hypoglycemic, hypotensive/hypotonic and other effects of drug overdose. Reduction to complete discontinuation of the previous prescribed symptomatic medications in Main group were as a result of the weight loss treatment, and we had no intention to stop taking the medications.

Overweight is a biological burden for the body, consuming additional immunological, antitoxic, and trophic functions and excretions^98^. The restricted diet (ANADETO) is considered to facilitate lipid metabolism and reduce fat mass, which could lead to suppression of inflammation^64,99^ and the enhancement of the immune response^100,101^, as well as the improvement of glucose/lipid metabolism and liver functions^35,45^.

Most of the body’s metabolism is expended on the nutritional (digestive) and reproductive systems^102^. Hyperactive sex lowers total body energy and shortens lifespan^103^. Sexual intercourse reduces the level of reproductive hormones. Each time after sexual intercourse, the body makes additional efforts to restore the supply of reproductive hormones^104,105^. People after sexual intercourse need rest due to a relative decrease in performance and mental abilities. Therefore, in Main group during the ANADETO period, we recommended sexual self-restraint.

The ability to accumulate adipose tissue is one of the most important adaptive mechanisms for survival, but we now observe a steady increase in obesity-related diseases^106,107^.

The fast weight loss method, including calorie restriction and walking, may be a safe, well-tolerated, and acceptable therapeutic option for patients with COVID-19 comorbid with the NCDs.

### Limitations

The strengths of this study are that a restricted diet and weight loss had a positive effect on patients with COVID-19 comorbid with NCDs in a prospective clinical trial. The limitations of this study are that it was a single-center study, it was short in duration, it did not include T-cell/B-cell subpopulation data, it was a nonrandomized trial, and there compares different treatment groups medication group vs medication + weight loss group. Further randomized controlled trials with long-term follow-ups are needed to confirm and extend the results of the study.

## Conclusions

Thus, the restriction diet improved clinical and laboratory/instrumental data on inflammation; glucose/lipid metabolism, systolic/diastolic BPs, and NASH biochemical outcomes, reactive oxygen species; and allowed to stop taking medications in patients with COVID-19 comorbid T2D/hypertension/NASH; and the fast weight loss treatment may be recommended as an alternative to traditional medical treatment. The fast weight loss was achieved due to a decrease in fat mass.

## Data Availability

The data are available from the authors upon reasonable request and with permission of the Ministry of Education and Science of the Republic of Kazakhstan. Those wishing to request the study data should contact Principal Investigator of a research grant: Dr. Oshakbayev Kuat (Email: kuat.oshakbayev@umc.org.kz, phone + 77013999394).

## Abbreviations

ALT: Alanine aminotransferase
ANADETO: ‘Analimentary detoxication’ weight loss method
AOPP: Advanced oxidation protein products
ARD: Acute respiratory disease
AST: Aspartate aminotransferase
BMI: Body mass index
BP: Blood pressure
CT: Computed tomography
COVID-19: SARS-CoV-2, a virus that causes the disease known as COVID-19
HbA1c: Glycosylated hemoglobin A1c
HDL: High-density lipoprotein
HOMA-IR: Homeostasis Model Assessment for Insulin Resistance
M ± SEM: Mean ± standard error of the mean
MDA: Malondialdehyde
NASH: Nonalcoholic steatohepatitis
NCDs: Noncommunicable chronic diseases
ROS: Reactive oxygen species
SOD: Superoxide dismutase
T2D: Type 2 diabetes mellitus
